# Draft Genome Sequence of Legionella pneumophila subsp. *pneumophila* Strain DSM 25199

**DOI:** 10.1128/mra.00048-23

**Published:** 2023-04-26

**Authors:** Neda Nezam-Abadi, Christopher J. R. Turkington, Lorraine A. Draper, Andrey N. Shkoporov, R. Paul Ross, Colin Hill

**Affiliations:** a APC Microbiome Ireland and School of Microbiology, University College Cork, Cork, Ireland; Loyola University Chicago

## Abstract

Here, we report the 3,426,844-bp draft genome sequence of Legionella pneumophila subsp. *pneumophila* strain DSM 25199, a serogroup 1 strain of L. pneumophila. The assembly consists of 24 contigs with an *N*_50_ of 300,843 bp.

## ANNOUNCEMENT

Legionella pneumophila is the main causative agent of Legionnaires’ disease, a severe pneumonia-like condition, and is capable of producing a flulike condition termed Pontiac fever (1). Serogroup 1 isolates are responsible for ~83% of culture-confirmed cases of L. pneumophila in the European Union and European Economic Area (2). Despite their association with this important disease, few L. pneumophila isolates exist within public culture collections for researchers to study (~35 L. pneumophila isolates are in the American Type Culture Collection [ATCC], German Collection of Microorganisms and Cell Cultures [DMSZ], and National Collection of Type Cultures [NCTC] at the time of writing), and many of these have no associated genomic data. As it is important that those isolates that are available in public collections also have readily accessible genomic information, we present the draft genome sequence of L. pneumophila subsp. *pneumophila* strain DSM 25199 (also known as Benidorm 030 E), a serogroup 1 strain of L. pneumophila available in public culture collections (ATCC 43108, DSM 25199, and NCTC 12006). Although there are no data available on the origin of this specific isolate (3), others belonging to the Benidorm 030 E subtype have been identified in instances of Legionnaires’ disease (4, 5) and within environmental samples (6).

L. pneumophila DSM 25199 was purchased from DSMZ. For genomic DNA isolation, L. pneumophila DSM 25199 was cultured on Buffered charcoal yeast extract α-ketoglutarate (BCYE-α) agar and incubated at 37°C for 48 h. During incubation, the plate was inside a closed plastic container with a damp paper towel to produce humid conditions. Cells from an overnight incubation at 37°C in Buffered yeast extract α-ketogluterate (BYE-α) broth were then used for DNA extraction via the GenElute bacterial genomic DNA (gDNA) kit (catalog no. NA2110-1KT; Merck) as per the manufacturer’s instructions. The bacterial gDNA concentration was measured using an Invitrogen Qubit 3.0 fluorometer and the Qubit double-stranded DNA (dsDNA) BR kit. Following extraction, gDNA was sent to Novogene (Cambridge, UK) for sequencing, where DNA was fragmented and then library prepped using the NEBNext Ultra DNA library prep kit and subjected to 2 × 150-bp paired-end Illumina sequencing.

A total of 6,857,180 reads were obtained from the genome sequencing. Reads were trimmed and filtered for quality using fastp v0.23.2 (7) with the following parameters: --length_required 60 --detect_adapter_for_pe --cut_front --cut_tail --cut_window_size 4 --cut_mean_quality 20 --trim_front1 10. Sequences corresponding to the Illumina phiX spike were then removed using BBDuk from the BBMap suite (v39.01; https://sourceforge.net/projects/bbmap/; with parameters k=31 hdist=1). Read quality throughout this process was assessed using FastQC v0.11.9 (8) and MultiQC v1.13 (9). The 6,838,360 paired-end reads remaining after trimming and filtering were then used as input for *de novo* genome assembly using SPAdes v3.15.5 (10) in “--isolate” mode with default parameters. Following assembly, short contigs (<500 bp) were removed, leaving a final draft genome assembly of 24 contigs totaling 3,426,844 bp (*N*_50_, 300,843 bp) with a G+C content of 38.3% and estimated average depth of ~237×. Assembly quality was assessed using CheckM v1.2.2 (11) and found to have a completeness of 100% and contamination of 0.19%. Genome annotation was achieved using Bakta v1.6.1 (12), which predicted a total of 3,044 coding sequences, 42 tRNAs, 27 noncoding RNAs (ncRNAs), 13 pseudogenes, and 3 rRNAs. Taxonomic classification as L. pneumophila was confirmed using GTDB-Tk v2.1.1 (13). Prophage prediction using PhiSpy v4.2.21 (14) and hafeZ v1.0.3 (15) and plasmid identification using RFPlasmid v0.0.18 (16) did not identify any such elements in the assembly.

### Data availability.

The annotated draft genome assembly of L. pneumophila DSM 25199 is available in GenBank under accession no. JAQIUR000000000 as part of BioProject accession no. PRJNA923147. The raw reads are deposited in the Sequence Read Archive (SRA) under accession no. SRR23071207. The code and instructions to reproduce this analysis are available online at GitHub (https://github.com/Chrisjrt/Nezam-Abadi_DSM21599_genome_MRA_2023).

## References

[1] Cordes LG, Fraser DW. 1980. Legionellosis: Legionnaires’ disease; Pontiac fever. Med Clin North Am 64:395–416. doi:10.1016/s0025-7125(16)31600-5. 6993807

[2] European Centre for Disease Prevention and Control. 2022. Legionnaires’ disease. *In* Annual epidemiological report for 2020. European Centre for Disease Prevention and Control, Solna, Sweden.

[3] Reimer LC, Sardà Carbasse J, Koblitz J, Ebeling C, Podstawka A, Overmann J. 2022. BacDive in 2022: the knowledge base for standardized bacterial and archaeal data. Nucleic Acids Res 50:D741–D746. doi:10.1093/nar/gkab961. 34718743 PMC8728306

[4] Stout JE, Joly J, Para M, Plouffe J, Ciesielski C, Blaser MJ, Yu VL. 1988. Comparison of molecular methods for subtyping patients and epidemiologically linked environmental isolates of Legionella pneumophila. J Infect Dis 157:486–495. doi:10.1093/infdis/157.3.486. 3343523

[5] Pelaz C, Martín-Bourgon C. 1993. Characterization of clinical and environmental isolates of Legionella associated with outbreaks and study of the infection sources. Enferm Infecc Microbiol Clin 11:359–365. (In Spanish.) 8399473

[6] Veríssimo A, Marrão G, da Silva FG, da Costa MS. 1991. Distribution of Legionella spp. in hydrothermal areas in continental Portugal and the island of São Miguel, Azores. Appl Environ Microbiol 57:2921–2927. doi:10.1128/aem.57.10.2921-2927.1991. 1746954 PMC183898

[7] Chen S, Zhou Y, Chen Y, Gu J. 2018. fastp: an ultra-fast all-in-one FASTQ preprocessor. Bioinformatics 34:i884–i890. doi:10.1093/bioinformatics/bty560. 30423086 PMC6129281

[8] Andrews S. 2010. FastQC: a quality control tool for high throughput sequence data. Babraham Bioinformatics, Babraham Institute, Cambridge, United UK.

[9] Ewels P, Magnusson M, Lundin S, Käller M. 2016. MultiQC: summarize analysis results for multiple tools and samples in a single report. Bioinformatics 32:3047–3048. doi:10.1093/bioinformatics/btw354. 27312411 PMC5039924

[10] Prjibelski A, Antipov D, Meleshko D, Lapidus A, Korobeynikov A. 2020. Using SPAdes de novo assembler. Curr Protoc Bioinformatics 70:e102. doi:10.1002/cpbi.102. 32559359

[11] Parks DH, Imelfort M, Skennerton CT, Hugenholtz P, Tyson GW. 2015. CheckM: assessing the quality of microbial genomes recovered from isolates, single cells, and metagenomes. Genome Res 25:1043–1055. doi:10.1101/gr.186072.114. 25977477 PMC4484387

[12] Schwengers O, Jelonek L, Dieckmann MA, Beyvers S, Blom J, Goesmann A. 2021. Bakta: rapid and standardized annotation of bacterial genomes via alignment-free sequence identification. Microb Genom 7:e000685. doi:10.1099/mgen.0.000685. PMC8743544 34739369

[13] Chaumeil P-A, Mussig AJ, Hugenholtz P, Parks DH. 2022. GTDB-Tk v2: memory friendly classification with the genome taxonomy database. Bioinformatics 38:5315–5316. doi:10.1093/bioinformatics/btac672. 36218463 PMC9710552

[14] Akhter S, Aziz RK, Edwards RA. 2012. PhiSpy: a novel algorithm for finding prophages in bacterial genomes that combines similarity- and composition-based strategies. Nucleic Acids Res 40:e126. doi:10.1093/nar/gks406. 22584627 PMC3439882

[15] Turkington CJR, Abadi NN, Edwards RA, Grasis JA. 2021. hafeZ: active prophage identification through read mapping. bioRxiv. doi:10.1101/2021.07.21.453177.

[16] van der Graaf-van Bloois L, Wagenaar JA, Zomer ALY. 2021. RFPlasmid: predicting plasmid sequences from short-read assembly data using machine learning. Microbial Genomics 7:e000683. doi:10.1099/mgen.0.000683. PMC8743549 34846288

